# Dialysis in Africa: the need for evidence-informed decision making

**DOI:** 10.1016/S2214-109X(20)30058-9

**Published:** 2020-04-01

**Authors:** Liam Crosby, Peter Baker, Peter Hangoma, Edwine Barasa, Vida Hamidi, Kalipso Chalkidou

**Affiliations:** Institute of Epidemiology and Healthcare, University College London, London WC1E 7HB, UK; Imperial College London, Medical School, St Mary’s Campus, London, UK; Centre for Global Development in Europe, London, UK; School of Public Health, School of Health Sciences, Ridgeway Campus, University of Zambia, Lusaka, Zambia; KEMRI-Wellcome Trust Research Programme, Nairobi, Kenya; Norwegian Institute of Public Health, Oslo, Norway; Imperial College London, Medical School, St Mary’s Campus, London, UK; Centre for Global Development in Europe, London, UK

Chronic kidney disease and acute kidney injury are leading causes of mortality and morbidity in sub-Saharan Africa. Chronic kidney disease accounts for 4 million disability-adjusted life-years (DALYs) lost across the continent annually, just behind diabetes (6·4 million DALYs).^1^ Increases in the prevalence of hypertension and type 2 diabetes are likely to increase the prevalence of chronic kidney disease in Africa, while high rates of intoxication and infections (such as malaria and HIV) drive acute kidney injury. Many people with acute kidney injury or advanced chronic kidney disease (ie, end-stage renal disease) require renal replacement therapy to remain alive.

The ethical and practical challenges of providing renal replacement therapy, including dialysis, are well documented, including financial feasibility, clinical optimisation, and questions of distributional equity.^2,3^ African countries have specific ethical and economic challenges when deciding about provision for people with or at risk of end-stage renal disease.^3^ Severe financial constraints make the high opportunity costs of dialysis particularly challenging. Transplants are not available in most African countries, making dialysis the only option for people with end-stage renal disease, yet restricting the benefits of short-term dialysis while awaiting transplant. The heavy medical and nonmedical financial burden for patients who are given dialysis mean that 59% of people in sub-Saharan Africa stop dialysis while it is still indicated.^4^ A systematic review highlighted very poor outcomes from end-stage renal disease in the region (eg, mortality of up to 80% among incident cases).^4^ Poor renal registry systems restrict our understanding of dialysis outcomes, and few high quality estimates of the costs of dialysis in Africa exist.^5^ Together, these factors mean the cost-effectiveness of renal replacement therapy, including different dialysis methods, is not clear in African settings

Nephrologists, at times supported by pharmaceutical and device manufacturers, have made strong cases for increasing access to dialysis for those with acute kidney injury and chronic kidney disease, including in Africa.^2,6^ Undoubtedly, strong rationales exist for extending access to dialysis: without it, patients with end-stage renal disease are at high risk of catastrophic health expenditure and death. At present, a very small proportion of those with end-stage renal disease in Africa are given dialysis,^7^ and rationing of dialysis in Africa is at times driven by socioeconomic rather than medical factors.

These arguments for extending dialysis provision side-step crucial questions of opportunity cost, distributional equity, and how to balance dialysis alongside other preventive or treatment services in health benefit packages. Dialysis has the potential to consume a disproportionate amount of healthcare resources. Providing dialysis to all who could benefit would be unaffordable in most sub-Saharan African countries. For example, the number of people requiring dialysis in one calendar year (we took 2010 estimates here as an example) has been conservatively estimated to be 19 000 in Kenya, 75 000 in Nigeria, and 6000 in Senegal.^7^ Given the estimated costs of providing haemodialysis in these countries,^5^ we calculated that the total costs of providing haemodialysis would be Int$1·7 billion in Kenya, $3·5 billion in Nigeria, and $450 million in Senegal, equivalent to 15–55% of total domestic governmental health expenditure (appendix pp 1–3). Because accuracy estimating the number of people who need dialysis is very challenging, we used alternative low estimates, sourced from the literature to do sensitivity analyses (appendix pp 1–3). From these analyses, we found that 8–37% of total domestic health expenditure would be used to address dialysis needs. In practice, and in keeping with cost-effectiveness studies from elsewhere,^8^ these estimates mean that the opportunity costs of wholesale dialysis expansion in Africa are too great to be easily justifiable from a population health perspective.

Therefore, decisions about how much to extend dialysis in Africa need to be made in the context of overall benefit package design, and alongside consideration of other components, such as preventative interventions or palliative care. Such considerations are particularly important in Africa where preventive efforts—tackling infections or hypertension and type 2 diabetes—could reduce the incidence of end-stage renal disease.

When considering an overall benefit package for those with or at risk of end-stage renal disease, policy makers need to clearly define the decision to be made, the criteria to be applied, and the process to be followed. Existing WHO guidance documents seek to inform policy makers what criteria should be applied in decisions about priority setting:^9^ all guidance documents agree that criteria about cost-effectiveness should be considered alongside criteria regarding the distribution of benefits. Most important is that the criteria, and the types of evidence that will be considered, are explicitly agreed, and that the process followed should be explicit, transparent, and accountable. If African countries do provide dialysis, they should seek to control costs by defining clear indications and auditing their use, implementing interventions for early detection, and seeking to reduce consumable costs—eg, by combining procurement arrangements across providers to aggregate demand, or addressing monopolies in supply.

The available evidence suggests that in Africa the costs of wholesale increases in access to dialysis are probably unaffordable for countries seeking sustainable health benefit packages. This challenge is particularly important in the context of tightening health-care budgets, other competing interventions, and slowing development assistance for health. Embedding institutionalised frameworks for priority setting that incorporate health technology assessment and transparent and fair processes is crucial to enabling funders to reach accountable decisions about dialysis and other interventions. Given the global nature of these challenges, countries may benefit from sharing their experience through networks such as the international Decision Support Initiative (iDSI).

Ultimately, African governments must define what criteria they will use for deciding about dialysisprovision, including clear consideration of trade-offs with other preventive or palliative interventions. They must consider dialysis as part of transparent, evidence-based, and fair processes to agree an overall package of interventions to be provided. Enhancing strategies for prevention and control of kidney disease rather than expensive dialysis might well warrant increased investment, leading to increased and more equitable benefits for health and poverty prevention.

## Supplementary Material

Supplementary appendix
