# Supplementary material for: Dialysis in Africa: the need for evidence-informed decision making
Source: Lancet Glob Health. Author manuscript; Available in PMC 2022 Sep 3. (PMC7613493; doi:10.1016/S2214-109X(20)30058-9)
Supplement: Supplementary appendix [file EMS153505-supplement-Supplementary_appendix.pdf]

THE LANCET
Global Health

Supplementary appendix

This appendix formed part of the original submission. We post it as supplied by the authors.

Supplement to: Crosby L, Baker P, Hangoma P, Barasa E, Hamidi V, Chalkidou K. Dialysis in Africa: the need for evidence-informed decision making. *Lancet Glob Health* 2020; **8:** e476–77.

Appendix– estimating the costs of providing dialysis in Kenya, Nigeria, Senegal.

1. The table overleaf shows the calculations made to estimate the costs of providing dialysis in these three countries.

2. Kenya, Nigeria and Senegal were chosen for this calculation, because estimates of the cost of dialysis in these countries are available in Mushi et al (2014). [1]

3. The 'conservative' estimate of the number of people in each country requiring dialysis in 2010 (***A***) were taken from modelling by Liyanage et al (2015, Supplementary Materials).[2] The 'conservative' number from Liyanage et al's paper was taken; for each country this was roughly half the size of their 'high' estimate. These estimates are based on modelling based on prevalence of dialysis provision in 16 high-income countries. They estimate how many people might require dialysis if countries delivered similar age-specific dialysis rates to 16 HICs, based on their demographic and epidemiological profiles. [Age-specific dialysis rates are presented in Table 1 of Liyanage et al's paper].

4. Sensitivity estimates of alternative numbers of people requiring dialysis (***A1***) were taken from a statement from the Cabinet Secretary for Health in Kenya;[3] and a statement from the President of the Nephrology Association in Nigeria.[4] No appropriate sensitivity estimate for Senegal was found.

5. The estimated annual costs of providing haemodialysis (***B***) were taken from Mushi et al's (2014) systematic review of the costs of dialysis. As three estimates were available for Nigeria, an average of the three costings studies was taken. These prices are for 2012. Estimates for 2010 were gained by adjusting for inflation (CPI 2010-2011 taken from the World Bank databank: i.e. 18.5% 26.1% and 4.7% over the two years in each of Kenya, Nigeria and Senegal, respectively).

6. Estimates of domestic governmental health expenditure in each country (***F***) were calculated based on estimates of GDP (PPP, Int$) (D) taken from World Bank data;[5] and domestic government health expenditure as % of GDP in 2010 (E), taken from WHO.[6]

7. The estimated cost of providing dialysis to all who need (C) is given by a multiplication of the number of people requiring dialysis (A) and the cost of providing it for one year (B). The proportion of total governmental health expenditure that would be required for dialysis (G) is this estimated cost (C), divided by the total health expenditure for 2010 (F). Sensitivity estimates for the cost of providing dialysis (C1) as a proportion of all expenditure (G1) are based on the alternative estimates of numbers of people requiring dialysis (A1).

	A	A1	B	C	C1	D	E	F	G	G1
	Total requiring dialysis ('Conserva-tive estimate', 2010)	Total requiring dialysis – ***'Sensitivity estimates'***	Costs of haemo-dialysis per year	Total cost of providing dialysis		GDP (PPP, Int$)	Domestic general government health expenditure as % of GDP	Domestic Government health expend (PPP, Int$)	Proportion of government health expenditure that would be required for dialysis	
Source of estimate	*Liyanage et al 2015*[1]	*Kenyan CS for Health, 2017;*[3] *Uzukwu, 2018.*[4]	*Mushi et al 2014*[2] *(adjusted to 2010 prices)*	*(=A*B)*	***Sensitivity estimate*** *(=A1*B)*	*WB data, 2010*[5]	*WHO Global Health Expenditure database*[6]	*(=D*E)*	*(=C/F)*	***Sensitivity estimate*** *(=C1/F)*
**Kenya**	19,018	10,000	$14,211	$270,258,482	$142,106,679	$100,285,000,000	1·77%	$1,776,594,154	15·2%	8.0%
**Nigeria**	74,873	50,000	$26,126	$1,956,098,099	$1,306,277,362	$789,561,000,000	0·45%	$3,540,946,822	55·2%	36.9%
**Senegal**	6,051	N/A	$27,157	$164,324,492		$34,471,000,000	1·33%	$458,924,060	35·8%	

References:

1 L. Mushi, P. Marschall, S. Flessa (2015). The cost of dialysis in low and middle-income countries: a systematic review. BMC Health Serv Res, 15 (2015), p. 50
2 Liyanage T, Ninomiya T, Jha V, et al (2015). Worldwide access to treatment for end-stage kidney disease: a systematic review. Lancet. 2015 May 16;385(9981):1975-82
3 Kenyan Ministry of Health, 2017. Press release: “Government steps up fight against kidney diseases”. Available from: http://www.health.go.ke/government-steps-up-fight-against-kidney-diseases/
4 Uzukwu 2018. Issues of Chronic Kidney Disease (CKD) and End Stage Kidney Disease (ESKD) in Southeastern Nigeria: A Study of Patients on Dialysis
5 World Bank data. Accessed 16th January 2020 from https://data.worldbank.org/indicator/NY.GDP.MKTP.PP.CD
6 WHO Global Health Expenditure Database. Accessed 16th January 2020 from https://apps.who.int/nha/database
